# Synthesis of Polythiophene–Fullerene Hybrid Additives as Potential Compatibilizers of BHJ Active Layers

**DOI:** 10.3390/polym8120440

**Published:** 2016-12-18

**Authors:** Sofia Kakogianni, Aikaterini K. Andreopoulou, Joannis K. Kallitsis

**Affiliations:** 1 Department of Chemistry, University of Patras, University Campus, GR26504 Patras, Greece; skakogianni@upatras.gr (S.K.); j.kallitsis@upatras.gr (J.K.K.); 2 Foundation for Research and Technology Hellas/Institute of Chemical Engineering Sciences (FORTH/ICE-HT), GR26504 Patras, Greece

**Keywords:** *rr*-polythiophenes, perfluorophenyl functionalization, fullerene-polymer hybrids, P3HT:PCBM blends, organic photovoltaics (OPVs)

## Abstract

Perfluorophenyl functionalities have been introduced as *side* chain substituents onto regioregular poly(3-hexyl thiophene) (*rr*-P3HT), under various percentages. These functional groups were then converted to azides which were used to create polymeric hybrid materials with fullerene species, either C_60_ or C_70_. The P3HT–fullerene hybrids thus formed were thereafter evaluated as potential compatibilizers of BHJ active layers comprising P3HT and fullerene based acceptors. Therefore, a systematic investigation of the optical and morphological properties of the purified polymer–fullerene hybrid materials was performed, via different complementary techniques. Additionally, P3HT:PC_70_BM blends containing various percentages of the herein synthesized hybrid material comprising *rr*-P3HT and C_70_ were investigated via Transmission Electron Microscopy (TEM) in an effort to understand the effect of the hybrids as additives on the morphology and nanophase separation of this typically used active layer blend for OPVs.

## 1. Introduction

Bulk Heterojunction (BHJ) solar cell devices are widely investigated due to their potential to reach high efficiencies along with their potential of large area coverage and unique flexibility [1,2,3]. Current state of the art materials that form the bicontinuous interpenetrating network of the active layer are poly(3-hexylthiophene) (P3HT) and [6,6]-phenyl-C_61_-butyric acid methyl ester (PCBM), providing power conversion efficiencies (PCE) of above 6%, because of their thermal stability [4], high electron mobility [5,6] and especially because it is the current system of choice for printable over large areas organic photovoltaics (OPVs) [7,8,9,10]. Among others, decisive parameters that affect the device performance are the materials comprising the active layer, the morphology of the blend as well as the device architecture. In the direction of materials development, new polymeric electron donating materials with complex architectures and lower energy levels [11,12,13,14,15] compared to P3HT, and new electron acceptors based on fullerene [16,17,18] or even fully organic and polymeric ones [19,20,21], have been reported. On the other hand, efforts to control the morphology [22,23] of the active blend have been made using thermal annealing or solvent treatment of the blend resulting in significantly higher PCEs [1,4,24,25,26]. Besides the above-mentioned manipulation of the active layer, another approach that has been followed is the modification of the semiconducting polymer with functional groups that may lead to more complex architectures either co-polymeric or hybrid ones. More specifically, co-polymeric materials based on polythiophenes or other conjugated polymers that can interact with or bear fullerene species can provide better miscibility and tune the nanophase separation of the two plain blend components [27,28,29,30,31,32,33]. In some cases, the introduction of functional groups like hydroxyl or azide units, as *side* chain substituents or as *end* chain groups, not only controls but also stabilizes the morphology of the blended materials [34,35,36,37,38]. Another efficient way to control the morphology is the introduction into the active layer of small quantities of hybrid materials comprising the semiconducting polymer and a carbon nanostructure [39,40,41,42,43,44,45,46,47,48]. However, the synthesis of most semiconducting polymeric or copolymeric hybrids involves complicated and multistep synthetic procedures that affect the yield and the cost of the final materials. Moreover, in most such cases, a non-conjugated linkage between the semiconducting polymer and the carbon nanostructure has been employed preventing electronic interactions among the polymeric and fullerene parts of the hybrid materials. To overcome the above bottlenecks, new synthetic strategies involving the “direct” attachment of the two components, organic and fullerene ones, comprising the hybrid materials have been developed [49,50,51,52,53,54]. Working in this direction, we have recently reported a straightforward methodology for the development of hybrid semiconducting polymeric materials using the electron deficient perfluorophenyl functionality inserted either onto copolymeric materials or as *end* polymeric chain group [49,54]. Benefits of this methodology are the direct electronic interactions between the two components [50], the absence of extra aliphatic or other non-conjugated parts used as connection bridges which increase the volume of insulating material within the active layer as well as the use of the intermediate perfluorophenyl aromatic azides that are stable over time and ambient storage conditions.

In this present work, we focused on the introduction of perfluorophenyl functionalities as *side* chain substituents of *regioregular*-poly(3-hexyl thiophene) (*rr*-P3HT). Several ratios were attempted in order to investigate the influence of the *side* functionalization onto the properties of *rr*-P3HT. Thereafter, using our previously reported methodology [49,54], the perfluorophenyl functionalities were transformed into azides that performed a [3+2] cycloaddition reaction with C_60_ or C_70_ fullerene species to provide the desired hybrid materials. Extensive purification has been performed in all cases and the purified materials were characterized in respect to their optical and morphological properties. These particular hybrid materials are ideal candidates for the compatibilization and stabilization of P3HT:fullerene active layers used in BHJ polymer photovoltaics, since the introduction of the fullerene species directly along the polythiophene’s backbone ensures the hybrids’ incorporation in the nanophase created by the neat blend components potentially holding them in close proximity over time and temperature variations during device operation. In order to support the hybrids potentiality as compatibilizers, mixtures of typical P3HT:PC_70_BM active BHJ blends with various percentages of the C_70_ based hybrid material were investigated in respect to the effect of the additive on the morphology of the blend.

## 2. Materials and Methods

### 2.1. Materials

*rr*-P3HT was synthesized according to literature procedure [55]. Fullerene C_60_ (99.5%) was purchased from SES research. Carbon C_70_ (99.5%) was kindly provided by Prof. Kyriakos Porfyrakis of the Oxford Carbon Nanomaterials Group, Department of Materials, University of Oxford, UK. Tetrahydrofuran was purchased from Aldrich (Sigma-Aldrich Chemie GmbH, Taufkirchen, Germany) and was distilled with benzophenone and metallic sodium (THF_dry_). All the other solvents and reagents were purchased from Aldrich and used without further purification unless otherwise stated.

### 2.2. Instrumentation

^1^H, ^19^F, ^15^N Nuclear Magnetic Resonance (NMR) spectra were recorded on a Bruker Advance (Bruker BioSpin GmbH, Magnet Division, Karlsruhe, Germany) DPX 400.13, 376.5 and 40.55 MHz spectrometer, respectively, in CDCl_3_ containing TMS as internal standard.

Gel permeation chromatography (GPC) measurements were carried out using a Polymer Lab chromatographer (Agilent Technologies, Santa Clara, CA, USA) equipped with two PLgel 5 μm mixed columns and a UV detector, using CHCl_3_ as eluent with a flow rate of 1 mL/min at 25 °C, calibrated versus polystyrene standards.

Attenuated Total Reflectance (ATR) spectra were recorded on a “Bruker Optics’ Alpha-P Diamond ATR Spectrometer of Bruker Optics GmbH” (Ettlingen, Germany).

Thermogravimetric analysis (TGA) was carried out on ~8 mg samples contained in alumina crucibles in a Labsys TM TG apparatus of Setaram (Caluire, France) under nitrogen and at a heating rate of 10 °C/min.

UV-Vis spectra were recorded using a Hitachi U-1800 spectrophotometer (Hitachi High-Technologies Europe GmbH, Mannheim, Germany) Continuous wave photoluminescence was measured on a Perkin Elmer LS50B spectrofluorometer (Waltham, MA, USA). All UV-Vis and PL measurements were performed in air using quartz cuvettes and flat substrates for the examination of solutions and films, respectively.

The Blend Preparation involved the mixing of stock solutions of P3HT (10 mg·mL^−1^) and PC_70_BM (10 mg·mL^−1^) to prepare the P3HT:PC_70_BM 1:1 *w*/*w*. Afterwards, 0.125 and 0.625 mL of the [P3HT_0.6_-(P3HT-5F)_0.3_-(P3HT-5F-N-C_70_)_0.1_] (8 mg·mL^−1^) were added to prepare the 20% and the 50% blends with the hybrid polymer, respectively.

Transmission electron microscopy (TEM) measurements were performed on a JEOL JEM2100 (Peabody, MA, USA) operating at 200 kV. Sample preparation for TEM examination of the [P3HT_0.6_-(P3HT-5F)_0.4_], [P3HT_0.6_-(P3HT-5F)_0.2_-(P3HT-5F-N-C_60_)_0.2_], [P3HT_0.6_-(P3HT-5F)_0.3_-(P3HT-5F-N-C_70_)_0.1_] involved the preparation of dilute solutions of the samples in *o*-DCB and filtration through a 0.45 μm filter. For the hybrids a drop of the solution was placed on 3 mm carbon coated copper grids (Electron Microscopy Sciences) and the samples were dried in air for 2 days. For the blends examination via TEM, a drop of the blends solution was placed on 3 mm carbon coated copper grids (Electron Microscopy Sciences) and after 30 s the solvent was removed with filter paper.

### 2.3. Synthetic Procedures

#### 2.3.1. *Side* [P3HT_0.6_-(P3HT-Br)_0.4_] [56]

A 50 mL round bottom flask, equipped with a reflux condenser and a magnetic stirrer, was flamed under vacuum degassed and filled with argon. P3HT (500 mg, 3 mmol) was added and dissolved in 30 mL CHCl_3_ and the system was degassed and flushed with argon again. The flask was covered with aluminum foil and NBS (214 mg, 1.2 mmol) was added in three portions. The mixture was stirred for 22 h at room temperature. Then, ~15 mL Na_2_SO_3_ 5% aqueous solution were added to the system and the mixture was extracted with CHCl_3_ and deionized water. The organic phase was dried with anhydrous MgSO_4_, filtrated and rotary evaporated. The obtained solid was dried under vacuum at 40 °C. Bromination ratio calculated from ^1^H NMR: 40%, yield: 92% ^1^H NMR (δ_H_; CDCl3; Me_4_Si): 7.00(broad, 1H), 2.80(broad, 2H), 2.64(broad, 2H–Br substituted thiophene), 1.65(broad, 4H), 1.53–1.22(broad, 12H), 0.88(broad, 6H).

The same procedure was used for all other ratios.

#### 2.3.2. *Side* [P3HT_0.6_-(P3HT-5F)_0.4_]

A 100 mL round bottom flask, equipped with a reflux condenser and a magnetic stirrer, was degassed (flamed under vacuum) and filled with argon. *Side* [P3HT_0.6_-(P3HT-Br)_0.4_] (400 mg, 0.81 mmol), pentafluorophenyl boronic acid (205 mg, 1 mmol) and Pd(PPh_3_)_4_ (30 mg, 0.02 mmol) were dissolved in 20 mL toluene and then Na_2_CO_3_ (1.20 mL, 2.42 mmol) in 2 M aqueous were added. The mixture was degassed and flushed with argon again and refluxed for 72 h. Then, the mixture was filtered in order to remove the catalyst and the solvent was rotary evaporated. The obtained solid was washed several times with MeOH and deionized water. The final solid was dried under vacuum at 50 °C. Yield: 99% ^1^H NMR (δ_H_; CDCl3; Me4Si): 7.00(broad, 1H), 2.80(broad, 2H), 2.64(broad, 2H), 1.65(broad, 4H), 1.53–1.22(broad, 12H), 0.88(broad, 6H), ^19^F NMR (δ_F_; CDCl3; Me4Si): −139.10, −150.91, −159.94.

#### 2.3.3. *Side* [P3HT_0.6_-(P3HT-5F)_0.2_-(P3HT-5F-N_3_)_0.2_]

A 50 mL round bottom flask with a reflux condenser and a magnetic stirrer was degassed and filled with argon. *Side* [P3HT_0.6_-(P3HT-5F)_0.4_] (400 mg, 1.72 mmol) was dissolved in 35 mL THF (dry) and NaN_3_ (22 mg, 0.34 mmol) was added. The mixture was degassed and flushed with argon again and heated at 40 °C for 48 h. The reaction mixture was then precipitated into a mixture of deionized water:methanol (1:1). The desired azide was obtained after filtration and the solid dried under vacuum at 30 °C for 24 h. Yield: 96% ^1^H NMR (δ_H_; CDCl3; Me4Si): 7.00(broad, 1H), 2.80(broad, 2H), 2.64(broad, 2H), 1.65(broad, 4H), 1.53–1.22(broad, 12H), 0.88(broad, 6H), ^19^F NMR (δ_F_; CDCl3; Me4Si): −135.76, −136.36, ^15^N NMR (δ_N_; CDCl3; Me4Si): 233.84, 51.73.

The same procedure was used for the side [P3HT_0.6_-(P3HT-5F)_0.3_-(P3HT-5F-N_3_)_0.1_].

#### 2.3.4. *Side* [P3HT_0.6_-(P3HT-5F)_0.2_-(P3HT-5F-N-C_60_)_0.2_]

A 50 mL round bottom flask, equipped with a reflux condenser and a magnetic stirrer, was degassed (flamed under vacuum) and filled with argon. *Side* [P3HT_0.6_-(P3HT-5F-N_3_)_0.4_] (150 mg), C_60_ (87 mg, 0.12 mmol) and 60 mL *o*-DCB were added and the system was degassed and flushed with argon again. The reaction mixture was stirred at 140 °C for 72 h. After evaporation of the solvent, the solid was stirred in toluene for 72 h in order to remove the unreacted C_60_. The mixture was filtrated and the solid was evaluated via Thin Layer Chromatography (TLC), using toluene as the mobile phase on silica gel plates, and ATR for traces of unreacted C_60_ and if necessary the solid was again stirred in toluene. The final purified hybrid material was dried under vacuum at 50 °C overnight affording 100.00 mg of the hybrid C_60_ based polymer.

#### 2.3.5. *Side* [P3HT_0.6_-(P3HT-5F)_0.3_-(P3HT-5F-N-C_70_)_0.1_]

A 50 mL round bottom flask, equipped with a reflux condenser and a magnetic stirrer, was degassed (flamed under vacuum) and filled with argon. *Side* [P3HT_0.6_-(P3HT-5F-N_3_)_0.4_] (150 mg), C_70_ (61 mg, 0.093 mmol) and 50 mL *o*-DCB were added and the system was degassed and flushed with argon again. The reaction mixture was stirred at 140 °C for 72 h. After evaporation of the solvent, the solid was stirred in toluene for 72 h in order to remove the unreacted C_70_. The mixture was filtrated and the solid was evaluated via TLC, using toluene as the mobile phase on silica gel plates, and ATR for traces of unreacted C_70_ in which case the solid was again stirred in toluene. The final purified material was dried under vacuum at 50 °C overnight affording 115.00 mg of the hybrid C_70_ based polymer.

## 3. Results and Discussion

### 3.1. Synthesis of Side-Br P3HTs

**Scheme 1:**
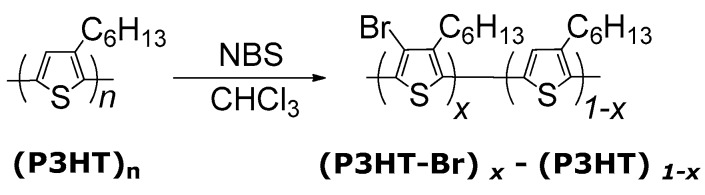
Synthetic procedure of *side* functionalization of *rr-*P3HT.

**Figure 1:**
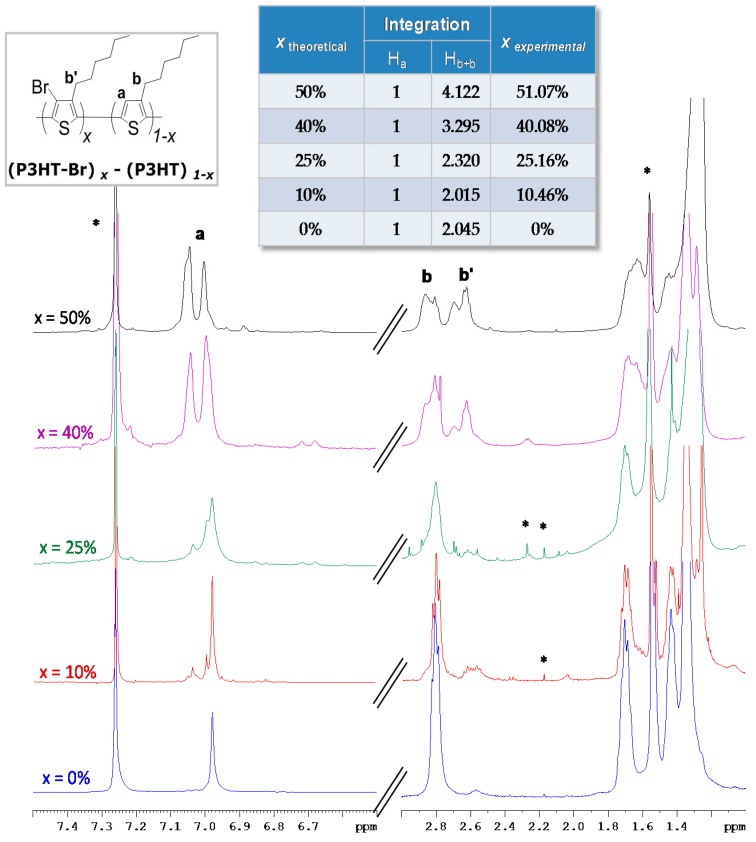
^1^H NMR spectra of the various *side* bromine functionalized P3HTs, in CDCl_3_ (the asterisks indicate solvent traces).

**Figure 2:**
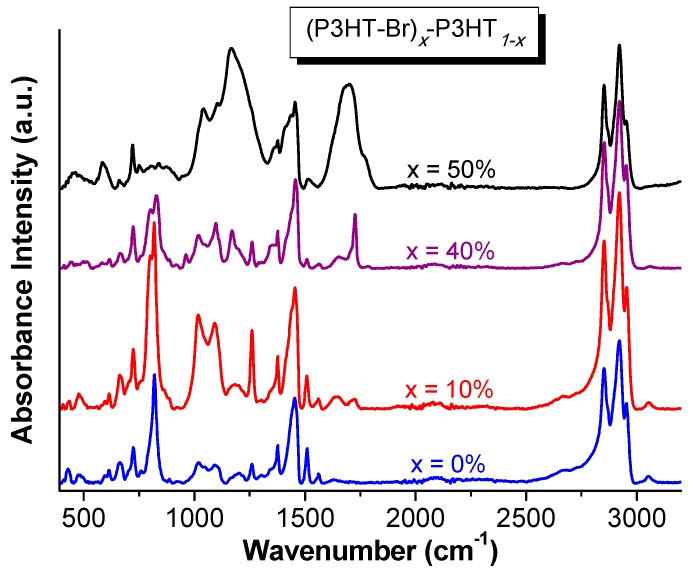
ATR spectra of an unfunctionalized P3HT and various ratios of *side* bromine-functionalized P3HTs.

Typical GRIM polymerization conditions of 2,5-dibromo-3-hexyl-thiophene in freshly distilled THF using MeMgCl as the Grignard reagent and NiCl_2_(dppp) as catalyst were used to prepare the initial *rr*-P3HT [55]. The crude polymer was obtained after precipitation in methanol and was further purified via Soxhlet extractions using methanol, *n*-hexane and chloroform as fractionation solvents. For this work, we used only the chloroform fraction of the *rr*-P3HT with a corresponding Mn = 28,500, PDI = 1.2. For the side functionalization of *rr*-P3HT, bromination at the 4-position of the thiophene ring was performed [41,56,57,58,59] (Scheme 1). Various equivalents of the brominating reagent, *N*-Bromosuccinimide (NBS), in respect to the thiophene repeating units were used to achieve different bromination degrees of the polymeric backbone. The rate of bromine-functionalization is calculated from the ^1^H NMR of the *side*-[P3HT-(P3HT-Br)] (Figure 1). The appearance of a broadened bimodal peak at ~7–7.2 ppm and of two peaks at ~2.8–2.6 ppm is attributed to the successful bromination of the thiophene ring at 4-position and also the partial disruption of the regioregularity of the P3HT’s backbone. More specifically, the insertion of the bromine atom at the 4-position of the thiophene ring causes the stepwise reduction of the aromatic hydrogens of P3HT initially located at ~7 ppm and a stepwise shift of the α-methylene hydrogen peak of the hexyl group from 2.8 to ~2.6 ppm. The initial *rr*-P3HT shows a peak at 2.8 ppm corresponding to the α-methylene protons of the hexyl aliphatic chains at the 3-position of the thiophene ring connected in a head-to-tail mode. After bromination at the 4-position of the thiophene ring, via electrophilic substitution affording the *side*-[P3HT-(P3HT-Br)] polymers (Scheme 1), a new broad proton signal at about 2.6 ppm appears attributed to the α-methylene protons of those hexyl chains attached to the partially brominated thienyl ring. This chemical shift is down-field shifted compared to that of their initial counterparts, indicating the influence of the bromine as an electron-attracting group on the α-methylene protons. From the integral ratio of the peaks at 2.8–2.6 ppm to the one at ~7 ppm, the bromine substitution ratio in the *side*-[P3HT-(P3HT-Br)] polymers is calculated. In the ATR spectra (Figure 2), it is observed that as the ratio of the bromine-functionalization increases the characteristic peaks of the *rr*-P3HT are broadened, compared to the unfunctionalized *rr*-P3HT. The appearance of two new peaks at ~1600 and ~1720 cm^−1^, respectively, is attributed to the successful bromination of the thiophene ring at the 4-position [41].

**Scheme 2:**
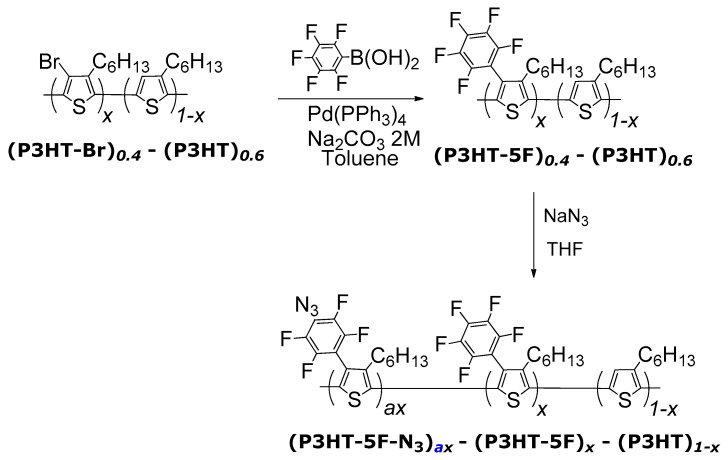
Synthetic procedure of *side* perfluorophenyl P3HT and their azide transformation; *a* indicates the azide functionalization degree.

**Figure 3:**
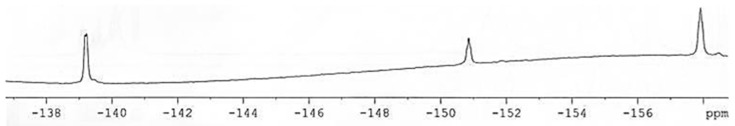
^19^F NMR spectra (in CDCl_3_) of the P3HT_0.6_-(P3HT-5F)_0.4_.

**Figure 4:**
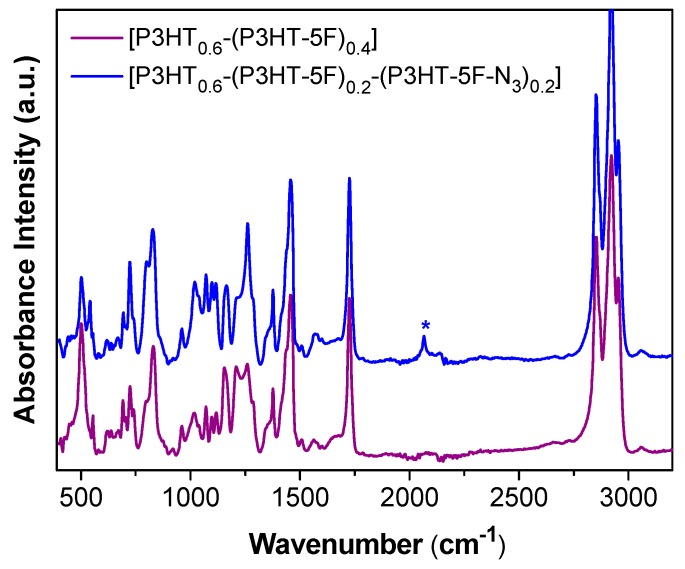
ATR spectra of the [P3HT_0.6_-(P3HT-5F)_0.4_] and azide [P3HT_0.6_-(P3HT-5F)_0.2_-(P3HT-5F-N_3_)_0.2_] (the asterisk indicates the azide functionality).

**Figure 5:**
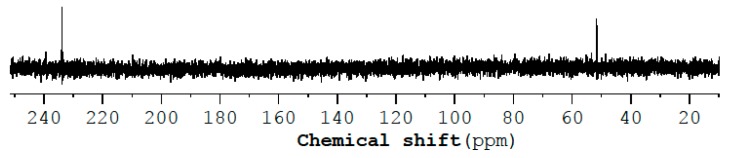
^15^N NMR spectra of the *side* [P3HT_0.6_-(P3HT-5F)_0.3_-P3HT-5F-N_3_)_0.1_], in CDCl_3_.

As it is known from previous literature findings on the side functionalization of *rr*-P3HT [41,56,57,58,59], high levels of functionalization at the 4-position of the thiophene ring affect the planarity along the polymer’s backbone and also disrupt the effective conjugation length of P3HT. Additionally, high functionalization degrees with fullerene species along the *rr*-P3HT backbone, which is our herein ultimate target, were inevitably going to provide hybrid materials with poor solubility in common organic solvents. Thus, and in order to prepare processable hybrid materials, but with adequate functionalization levels and with efficient number of *side*-fullerene units to act as compatibilizers of the neat components’ blends, we chose the 40% *side* bromine-functionalized P3HT for further studies. Therefore, the *side rr*-[P3HT_0.6_-(P3HT-Br)_0.4_] reacted with an equivalent amount of perfluorophenyl boronic acid under Suzuki cross coupling conditions to form the respective *side* [P3HT_0.6_-(P3HT-5F)_0.4_] polymer (Scheme 2). The successful introduction of the perfluorophenyl ring at the side position of the P3HT’s polymeric backbone was proven through ^19^F NMR spectroscopy (Figure 3). The ^19^F NMR spectrum presents three peaks attributed to the three fluorine species of the perfluorinated ring. Afterwards, the perfluorophenyl groups of the P3HT_0.6_-(P3HT-5F)_0.4_ polymer were transformed into azides using sodium azide in THF [38]. Various azidation degrees were employed affording materials with 40%, 20% or 10% overall theoretical azidation degrees (Scheme 2, Table 1) in order to be able thereafter to incorporate different amounts of fullerenes and eventually produce processable hybrid polymers with C_60_ or C_70_. The successful azidation was confirmed using ATR and ^15^N NMR spectroscopies. The ATR spectrum of the 20% side perfluorophenyl azide showed a low intensity peak at 2064 cm^−1^ which demonstrates the azide formation (Figure 4), while for the 10% azide functionalized polymer the azide peak was not clearly observed due to the low ratio of the azide functionality over the total polymer. On the other hand, ^15^N NMR spectroscopy, as shown in Figure 5, revealed two clear peaks owing to the two nitrogen species of the azide functionality even for the 10% azide functionalized polymer.

### 3.2. Synthesis of Side-P3HT-(P3HT-5F) Hybrids

**Scheme 3:**
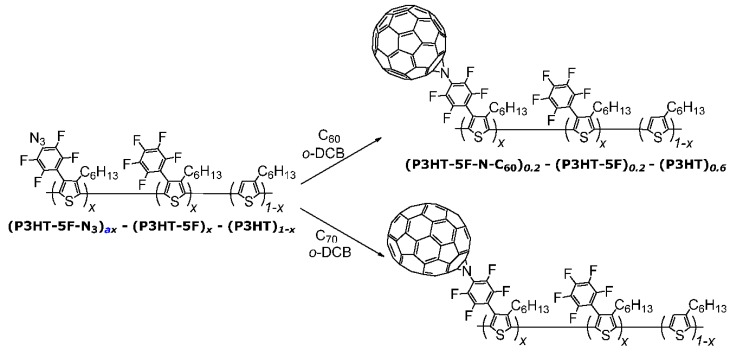
Synthetic procedure of *side*-[P3HT_0.6_-(P3HT-5F)_0.4_] hybrids with C_60_ or C_70_ fullerenes.

**Figure 6:**
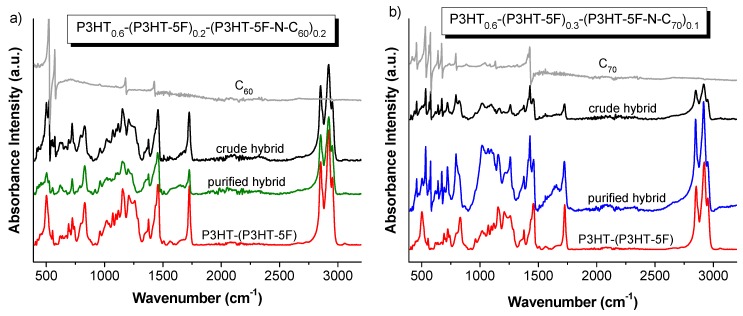
ATR spectra of: (**a**) C_60_; and (**b**) C_70_ based *side* [(P3HT_0.6_-P3HT-5F)_0.4_] hybrids, in comparison with the initial P3HT-(P3HT-5F) and the plain fullerenes, respectively.

The *side* P3HT-perfluorophenyl azides have been used to prepare side hybrid semiconducting polymers with either C_60_ or C_70_ fullerenes. When the *side* [P3HT_0.6_-(P3HT-5F-N_3_)_0.4_] reacted with an equivalent amount of C_60_ to the azide content, the final hybrid polymer was insoluble in common organic solvents. In order to avoid such insolubility problems, the *side* [P3HT_0.6_-(P3HT-5F)_0.2_-(P3HT-5F-N_3_)_0.2_] reacted with C_60_ fullerene while the *side* [P3HT_0.6_-(P3HT-5F)_0.3_-(P3HT-5F-N_3_)_0.1_] reacted with C_70_ providing the desired hybrid semiconducting polymers (Scheme 3) and leading to processable hybrid materials. After the [3+2] cycloaddition reaction, the *side* [P3HT-(P3HT-5F)]-hybrids with fullerenes were thoroughly washed several times with toluene to remove any unreacted fullerene species. A thorough ATR examination was performed for both hybrid materials after each subsequent washing step. When no further reduction of the fullerene peaks was noticed, we concluded that all unreacted traces of C_60_ or C_70_ were successfully removed (Figure 6).

**Figure 7:**
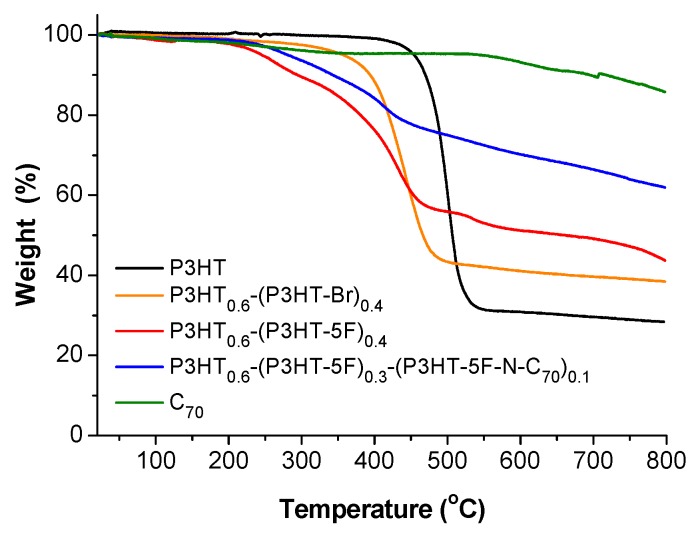
TGA thermograms of *rr*-P3HT, [P3HT_0.6_-(P3HT-Br)_0.4_], [P3HT_0.6_-(P3HT-5F)_0.4_], [P3HT_0.6_-(P3HT-5F)_0.3_-(P3HT-5F-N-C_70_)_0.1_] and C_70_ fullerene.

Thermogravimetric analysis (TGA) of the *side*-functionalized [P3HT_0.6_-(P3HT-5F)_0.4_] and the hybrid with fullerene C_70_ (Figure 7) revealed at 800 °C a 44% residue for the [P3HT_0.6_-(P3HT-5F)_0.4_] and a 62% residue for the [P3HT_0.6_-(P3HT-5F)_0.3_-(P3HT-5F-N-C_70_)_0.1_] hybrid, respectively. As expected, the hybrid polymer presented a higher residue due to the incorporation of the fullerene species.

### 3.3. Optical Properties

**Figure 8:**
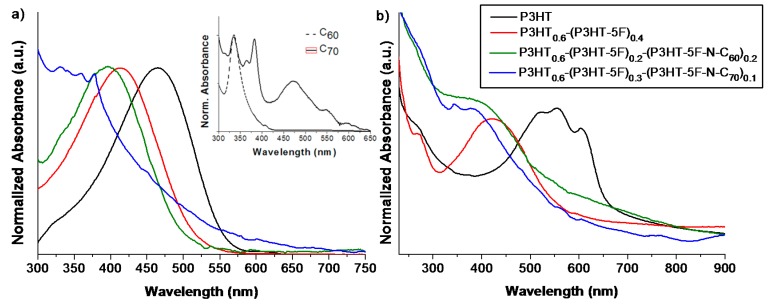
Normalized Absorption spectra in: (**a**) *o*-DCB solutions; and (**b**) film form of un functionalized P3HT, *side* functionalized [P3HT_0.6_-(P3HT-Br)_0.4_], [P3HT_0.6_-(P3HT-5F)_0.4_], [P3HT_0.6_-(P3HT-5F)_0.2_-(P3HT-5F-N-C_60_)_0.2_] and [P3HT_0.6_-(P3HT-5F)_0.3_-(P3HT-5F-N-C_70_)_0.1_], respectively. The inset in (**a**) shows the Normalized Absorption spectra of C_60_ and C_70_ in *o*-DCB solutions.

The optical properties of the synthesized hybrid materials compared to the initial *side* [P3HT_0.6_-(P3HT-5F)_0.4_] were investigated both in *o-*DCB solutions and in film form. The UV-Vis spectra of the initial *side*-[P3HT_0.6_-(P3HT-5F)_0.4_] both in *o*-DCB solution (Figure 8a) and in film form (Figure 8a) presented a blue shift compared to the unfunctionalized *rr*-P3HT. This phenomenon is attributed to the perturbation of the regioregularity of the P3HT backbone after the functionalization at the 4-position of the thiophene unit. The hybrid material [P3HT_0.6_-(P3HT-5F)_0.2_-(P3HT-5F-N-C_60_)_0.2_] presented an even greater blue-shift compared to its functionalized P3HT precursor, while the characteristic absorbance bands of the fullerene C_60_ were observed in solution as a shoulder at ~330 nm whereas, in film form, the absorption peak up to ~300 nm peak is attributed to the fullerenic part of the hybrid. On the other hand, the hybrid material [P3HT_0.6_-(P3HT-5F)_0.3_-(P3HT-5F-N-C_70_)_0.1_] clearly presented the characteristic absorbance bands of C_70_ fullerene both in solutions and in film form in the region 300–400 nm.

**Figure 9:**
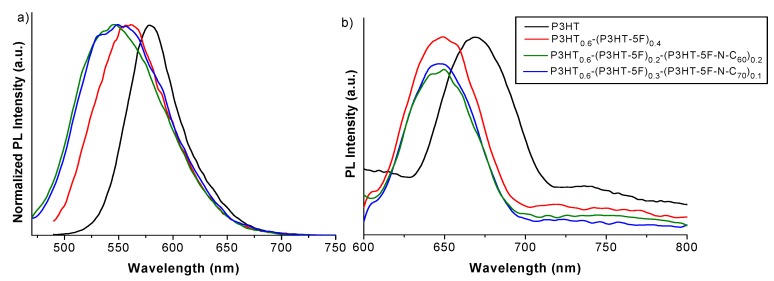
Photoluminescence spectra of: (**a**) *o*-DCB solutions; and (**b**) in film form of un functionalized *rr-*P3HT, *side* functionalized [P3HT_0.6_-(P3HT-5F)_0.4_], [P3HT_0.6_-(P3HT-5F)_0.2_-(P3HT-5F-N-C_60_)_0.2_] hybrid and [P3HT_0.6_-(P3HT-5F)_0.3_-(P3HT-5F-N-C_70_)_0.1_] hybrid, respectively.

The PL spectra of *side-*[P3HT_0.6_-(P3HT-5F)_0.4_] and its respective hybrids with C_60_ and C_70_ both in *o*-DCB solutions and in film form are shown in Figure 9. The *o*-DCB solutions of the hybrid materials, upon excitation at 470 nm, presented blue shifted spectra and broader photoluminescence curves in the region 500–650 nm, compared to the un-functionalized P3HT (Figure 9a). In film form, after excitation at the UV-Vis films maxima, the PL spectra followed the trend of the respective solutions also showing a blue shift compared to the neat *rr*-P3HT. A small quenching of the hybrids’ photoluminescence intensity in film form was observed, [P3HT_0.6_-(P3HT-5F)_0.2_-(P3HT-5F-N-C_60_)_0.2_] green-colored line and [P3HT_0.6_-(P3HT-5F)_0.3_-(P3HT-5F-N-C_70_)_0.1_] blue-colored line of Figure 9b, that is attributed to the electron accepting fullerene parts interacting electronically with the electron donating P3HT polymeric backbone and the perturbation of the P3HT’s backbone due to the side functionalization.

### 3.4. Morphology Characterization

**Figure 10:**
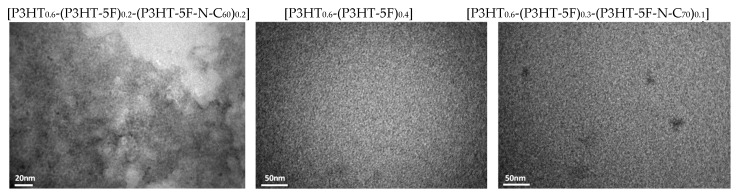
TEM images of the initial [P3HT_0.6_-(P3HT-5F)_0.4_] and its respective hybrids [P3HT_0.6_-(P3HT-5F)_0.2_-(P3HT-5F-N-C_60_)_0.2_] and [P3HT_0.6_-(P3HT-5F)_0.3_-(P3HT-5F-N-C_70_)_0.1_] without thermal annealing.

Thin film morphology of *side* perfluorophenyl functionalized [P3HT_0.6_-(P3HT-5F)_0.4_] and its respective hybrids with C_60_ and C_70_ was investigated using transmission electron spectroscopy (TEM) (Figure 10). The initial *side* [P3HT_0.6_-(P3HT-5F)_0.4_] formed a uniform thin film in the nanometer scale. The corresponding C_60_ based hybrid, [P3HT_0.6_-(P3HT-5F)_0.2_-(P3HT-5F-N-C_60_)_0.2_], formed films with large domains compared to the initial perfluorophenyl modified P3HT, with darker and brighter regions. In contrast, the C_70_ based hybrid polymer, [P3HT_0.6_-(P3HT-5F)_0.3_-(P3HT-5F-N-C_70_)_0.1_], presented a uniform nanophase separated thin film formation with well resolved small domains in analogy to its non hybrid initial polymer, [P3HT_0.6_-(P3HT-5F)_0.4_].

**Figure 11:**
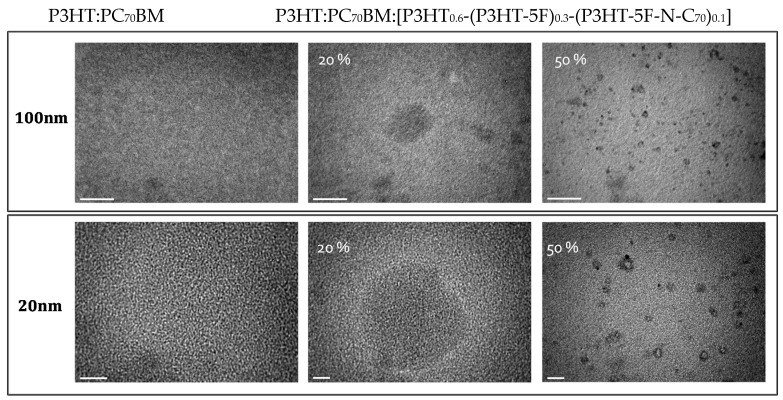
TEM images of a P3HT:PC_70_BM 1:1 *w*/*w* blend and of P3HT:PC_70_BM:[P3HT_0.6_-(P3HT-5F)_0.3_-(P3HT-5F-N-C_70_)_0.1_] blends with a 20 wt % and a 50 wt % of the [P3HT_0.6_-(P3HT-5F)_0.3_-(P3HT-5F-N-C_70_)_0.1_] hybrid, respectively. For the top images, the scale bars correspond to 100 nm while, for the bottom images, they correspond to 20 nm.

Moreover, a typical P3HT:PC_70_BM blend of 1:1 *w*/*w* ratio was employed to prepare two new blends with the [P3HT_0.6_-(P3HT-5F)_0.3_-(P3HT-5F-N-C_70_)_0.1_] hybrid polymer. The hybrid material was added at a 20 wt % and at a 50 wt % percent, respectively, over the total weight of the P3HT:PC_70_BM initial blend. The effect of the additive on the morphology of the initial blend was investigated via TEM microscopy. Figure 11 presents the respective TEM images in 100 nm and in 20 nm scale, of the initial blend and of the blends containing the hybrid polymer without any thermal or other solvent vapor treatment. The P3HT:PC_70_BM blend presented uniform thin film with nanophase separation. The morphology of the blend containing 20% of the side C_70_ based hybrid polymer did not present any differentiations, even at the 20 nm scale, showing the probable stabilization of the initial blends morphology. On the other hand, the blend with the 50% of the *side* C_70_ hybrid polymer formed more heterogeneous films in which darker domains are present.

## 4. Conclusions

In this work, modification in various ratios of the *rr*-P3HT backbone in order to introduce the perfluorophenyl functionality at the 4-position of the thiophene unit has been efficiently performed. Thereafter, and based on our previous reported methodology, the perfluorophenyl moieties transformed into azides, from which hybrid materials with fullerenes, C_60_ or C_70_, have been synthesized aiming to develop efficient compatibilizers and stabilizers of P3HT:fullerene active layers typically employed in large area flexible OPVs. The side-functionalized P3HTs and their respective hybrid polymers have been characterized in respect to their optical and morphological properties. In solutions and in film form, all the side-functionalized materials presented blue shifted absorbance curves due to the perturbation of regioregularity of polythiophene. Regarding their photoluminescence in film form, the hybrid materials presented moderate photoluminescence quenching. The C_70_ based hybrid polymer formed uniform nanophase separated thin films while the C_60_ based hybrid formed more heterogeneous thin films exhibiting larger domains. In order to investigate their applicability as potential compatibilizers of polythiophene:fullerene BHJ active layers, selected hybrids were evaluated for their influence on the morphology of a typical P3HT:PC_70_BM active layer blend. More specifically, when the percentage of the hybrid was low compared to the total blend weight ratio, the morphology was not affected. However, when the hybrid polymer was added at a higher percentage, the final blend presented irregularities and large domains were created throughout the blend.

Based on these findings it can be concluded that the particular “perfluorophenyl”-“azide”-“carbon nanostructure hybrid” methodology is conveniently applicable for the development of side hybrid semiconducting polymer electron donors allowing the insertion of fullerene species in the desired percentage. Such hybrid polymer–fullerene materials can be employed as additives of typical polymer-donor:fullerene-acceptor active layer blends for OPVs.

## Figures and Tables

**Table 1:** Azidation ratios of [P3HT_0.6_-(P3HT-5F)_0.4_] and the respective abbreviations of the functionalized polymers obtained.

Azide Functionalization Degree of (P3HT-5F-N_3_)_*ax*_-(P3HT-5F)_*x*_-(P3HT)_1−*x*_	Side azide
1	[P3HT_0.6_-(P3HT-5F-N_3_)_0.4_]
0.5	[P3HT_0.6_-(P3HT-5F)_0.2_-(P3HT-5F-N_3_)_0.2_]
0.25	[P3HT_0.6_-(P3HT-5F)_0.3_-(P3HT-5F-N_3_)_0.1_]
